# Process-Based Therapy as a Novel Treatment Approach and Framework for Classifying Psychopathology

**DOI:** 10.32872/cpe.13727

**Published:** 2024-03-28

**Authors:** Stefan G. Hofmann, Steven C. Hayes

**Affiliations:** 1 Translational Clinical Psychology Group, Department of Psychology, Philipps University of Marburg, Marburg, Germany; 2 Department of Psychology, University of Nevada, Reno, NV, USA

In a recent article, one of us co-authored a discussion paper comparing prominent classification frameworks (Rief et al., 2023). In the discussion, the article noted the following:

“PBT is primarily a treatment approach, while the systems perspective is a broader framework for understanding mental disorders. While PBT draws on the systems perspective to inform its understanding of mental disorders, it is primarily focused on developing and implementing novel interventions. The systems perspective, on the other hand, seeks to provide a comprehensive understanding of mental disorders that can inform the development of a wide range of future treatments” (p. 27).

We would like to correct and clarify these statements. In fact, PBT is *not* primarily a specific treatment approach, and it *does* seek a broader understanding of mental and behavioral health. In essence, PBT provides a different and more idiographic perspective on systems approaches to clinical science. It begins with an idiographic focus on how processes of change combine in complex networks and can best be altered case by case, which is extended to nomothetic principles if and only if doing so maintains or increases idiographic fit: what we term an “idionomic” approach.

As we noted in one of our first publications introducing PBT (Hofmann & Hayes, 2019), we contend that modern clinical science needs to focus on the following question: “What core biopsychosocial processes should be targeted with this client given this goal in this situation, and how can they most efficiently and effectively be changed?” (p. 38). Our proposed answer was an idionomic understanding of “the contextually specific use of evidence-based processes linked to evidence-based procedures to help solve the problems and promote the prosperity of particular people” (p. 38).

In the context of evolutionary science, adaptation or maladaptation is a function of variation, selection, and retention of biopsychosocial processes in given contexts. Any process can be helpful or hurtful depending on the person’s history, goals, or circumstances. Processes are often functionally interconnected, forming a complex network that may differ in degree of abstraction and complexity.

We contend that a broader and more functional approach to mental health will come by viewing psychopathology as a complex system – evolution gone awry within networks of biopsychosocial processes in the life trajectories of individuals, that may then be corrected with intervention. When such knowledge is extended in an idionomic fashion PBT argues it will provide a comprehensive understanding of mental disorders that can inform the development of a wide range of future treatments.

We hope this clarifies the distinguishing features of PBT and other frameworks discussed in the article by Rief et al. (2023).
